# Efficacy and safety of acupuncture therapy for COVID-19: A protocol for systematic review and meta-analysis: Retraction

**DOI:** 10.1097/MD.0000000000025226

**Published:** 2021-03-12

The article “Efficacy and safety of acupuncture therapy for COVID-19: A protocol for systematic review and meta-analysis”,^[1]^ which appeared in Volume 99, Issue 22 of Medicine, is being retracted due to self-duplication. An investigation into the paper and a second protocol published in Medicine^[2]^ with shared authors found the language and aim of both papers were similar enough to constitute duplication.
